# Elevated Serum Levels of Soluble CD30 in Ankylosing Spondylitis Patients and Its Association with Disease Severity-Related Parameters

**DOI:** 10.1155/2015/617282

**Published:** 2015-07-26

**Authors:** Rongfen Gao, Wei Sun, Yu Chen, Yuying Su, Chenqiong Wang, Lingli Dong

**Affiliations:** ^1^Department of Rheumatology and Immunology, Tongji Hospital, Huazhong University of Science and Technology, Wuhan 430030, China; ^2^Department of Stomatology, Union Hospital, Huazhong University of Science and Technology, Wuhan 430022, China

## Abstract

Soluble CD30 (sCD30), a transmembrane glycoprotein that belongs to the tumor necrosis factor receptor (TNFR) superfamily, has been shown to be associated with various pathological conditions. This study was designed to measure the levels of serum sCD30 in patients with ankylosing spondylitis (AS) and to evaluate the relationships between serum sCD30 levels and other disease severity-related indexes, including bath ankylosing spondylitis disease activity index (BASDAI), ankylosing spondylitis disease activity score (ASDAS), and bath ankylosing spondylitis functional index (BASFI). Our results demonstrated significantly elevated sCD30 levels in AS patients compared to healthy controls (HCs) with mean values of 32.0 ± 12.2 and 24.9 ± 8.0 ng/mL, respectively (*P* ^**^ = 0.007), suggesting a potential role of sCD30 in the pathogenesis of AS. However, no significant correlations of sCD30 with BASDAI, ASDAS, or BASFI were detected in our study (*P* > 0.05). Therefore, sCD30 cannot be used as a reliable marker for reflecting disease activity and functional ability of AS patients.

## 1. Introduction

Ankylosing spondylitis (AS) is a chronic inflammatory disorder that mainly affects the sacroiliac joints and axial skeleton of young males. The condition can lead to new bone formation and even disability if early diagnosis and treatment do not occur. The effects of environmental factors on genetically susceptible individuals are considered to be one possible mechanism for the pathogenesis of AS. It is known to be a highly genetic disease and there is a strong genetic association of MHC molecules with AS, especially human leukocyte antigen-B27 (HLA-B27) [1]. HLA-B27 is an MHC class I molecule that is highly expressed on antigen presenting cells. However, the exact roles of HLA-B27 in the pathogenesis of AS remain unclear, and several hypotheses have been proposed. For example, self or bacterial antigen peptides might be presented by HLA-B27 to CD8+ T cells, which would consequently lead to an aberrant immunological response. In addition, other non-HLA-B27 and non-MHC genes have also been shown to be associated with AS, including HLA-B60, HLA-B61, and the IL-1 gene cluster. Moreover, a recent study found that imbalances in subsets of T cell populations might be responsible for the pathogenesis of AS, including increased ratios of Th1/Th2 and Th17/Treg cells [2]. Abnormal functions of immune cells in patients with AS can lead to the upregulation of proinflammatory cytokines, including tumor necrosis factor-*α* (TNF-*α*), IL-6, soluble IL-2 receptor, IL-17, and IL-23 [1–3].

CD30 is a member of the TNF receptor superfamily and was originally recognized as a marker of Hodgkin and Reed-Sternberg cells in Hodgkin's lymphoma [4]. The protein is predominantly expressed on the surface of activated and memory T helper (Th) cells, rather than resting T and B cells. Subsequent studies have discovered that it is also present on other types of cells, including activated B cells, natural killer (NK) cells, dendritic cells [5], and neoplastic cells (such as myeloma or solid carcinoma cells) [6]. The CD30 ligand (CD30L) is also a member of the TNF superfamily and is expressed on activated T cells, resting B cells, monocytes, and granulocytes. Pleiotropic biological effects are induced on CD30+ cells after the ligation of CD30 with CD30L, including activation, proliferation, differentiation, and cell death in a cell type-dependent manner, respectively [7, 8]. It has been shown that the extramembranous region of CD30 is cleaved by a metalloprotease and released into the bloodstream after cell activation as a soluble protein (sCD30), and recent studies have explored the roles of the protein in various diseases, including malignant disorders, infectious diseases [4], rejection after organic transplantation [9], and autoimmune diseases [10–14]. Moreover, it has been proposed that sCD30 may serve as a diagnostic marker in various autoimmune disorders. However, the changes in sCD30 occurring in AS patients remain poorly understood. In this study, we assessed sCD30 serum levels in AS patients and assessed whether there is an association with severity-related indicators.

## 2. Material and Methods

### 2.1. Patient Characteristics

Thirty-five patients who fulfilled the modified New York criteria for AS [15] and had visited our department between 2012 and 2013 participated in this study. A questionnaire was used to record the basic and clinical information of patients, including age, sex, bath ankylosing spondylitis disease activity index (BASDAI), ankylosing spondylitis disease activity score (ASDAS), bath ankylosing spondylitis functional index (BASFI), erythrocyte sedimentation rate (ESR), and C-reactive protein (CRP). The scores of each scoring system ranged from 0 to 10. BASDAI and ASDAS were employed to evaluate the activity of AS, both of which are considered to be reliable indicators [16, 17]. Physical functions were assessed through BASFI, which is widely recognized for its strong reliability and construct validity [18]. The ESR was detected using the Westergren method while CRP measurements were taken by immunonephelometry using CRP reagents (BioSystems S.A, Spanish). Thirty-two age-matched healthy volunteers served as healthy controls (HCs). The exclusive criteria for AS patients were as follows: history of malignant cancers, infection, rheumatic diseases, or common diseases that presented obvious laboratory abnormalities. All AS patients enrolled in this study had not received biological agent therapies, such as a TNF-*α* inhibitor. HCs were volunteers who had no evidence of acute or chronic infectious disorders, autoimmune disease, or any other systemic condition. Written, informed consent was provided by each participant and the study was approved by the ethics committee of our university.

### 2.2. Determination of Serum sCD30 Levels

Peripheral blood was collected and processed by centrifugation. Serum was stored at −80°C until it was analyzed. Titers of sCD30 were determined using a commercial enzyme-linked immunosorbent assay (ELISA) kit according to the manufacturer's instructions (eBioscience).

### 2.3. Statistical Analysis

Data in this paper were expressed as mean value ± standard deviation (SD). For statistical analysis, an independent samples *t*-test was used to compare the sCD30 levels between the two groups in this study. Correlation analyses were carried out using Pearson's rank correlation test and *P* < 0.05 was considered statistically significant. All statistical analyses were conducted using Statistical Package for Social Sciences (SPSS) software for Windows.

## 3. Results and Discussion

### 3.1. Serum sCD30 Levels Are Notably Elevated in AS Patients but Do Not Correlate with Disease Severity-Related Parameters

**Figure 1:**
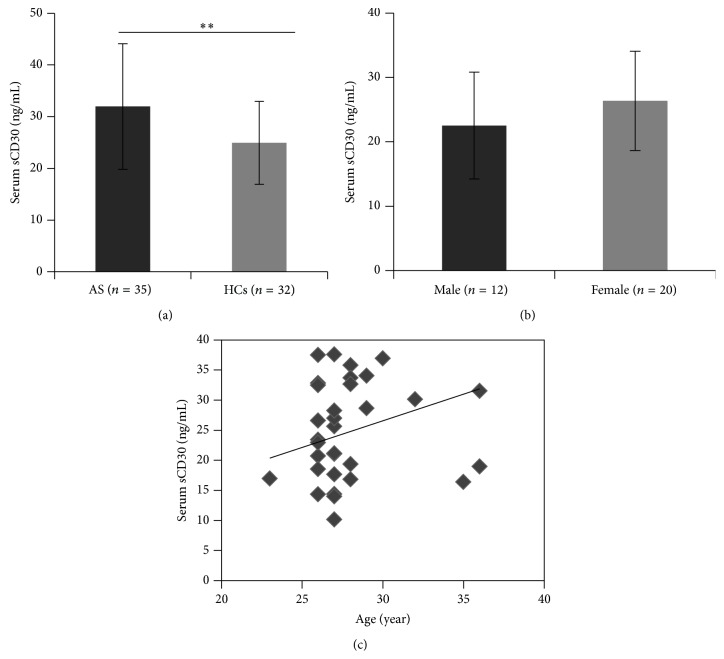
Serum levels of sCD30 in AS patients and healthy controls (HCs) were determined using a commercial enzyme-linked immunosorbent assay (ELISA) kit according to the manufacturer's instructions (eBioscience). (a) Serum sCD30 levels in 35 AS patients and 32 healthy controls; (b) serum sCD30 concentrations in healthy male and female individuals; (c) the correlation of serum sCD30 levels with age in HCs (*r* = 0.09, *P* = 0.63). ^**^ *P* < 0.01.

Our results revealed a statistically significant increase of serum sCD30 levels in AS patients compared to HCs (32.0 ± 12.2 and 24.9 ± 8.0 ng/mL, resp.; Figure 1(a)). We excluded patients who had previously undergone or were currently receiving treatment with biological agents; however, 3 cases (8.6%) stated that they had taken sulfasalazine for a short period of time (less than 3 months) and 1 case (2.9%) received glucocorticoid therapy 1 day prior to going on study (Table 1). The average serum sCD30 level for these 4 cases was 38.91 ± 8.87 ng/mL, hardly revealing a significant decrease. Moreover, there were no statistically significant differences between the 2 groups when these 4 cases were excluded from the analysis (data not shown). The small number of patients treated with disease modifying antirheumatic drugs (DMARDs) or glucocorticoids made it impossible to assess the effects of the drugs on serum sCD30 levels, and, therefore, a future prospective study is needed. Furthermore, serum sCD30 levels in male and female healthy controls were not statistically different (22.5 ± 8.3 and 26.4 ± 7.7 ng/mL, resp.; *P* > 0.05; Figure 1(b)). Unlike other studies showing significant correlations of sCD30 with disease severity indexes in several autoimmune disorders, our data indicated that serum sCD30 levels did not correlate with BASDAI (*r* = −0.11, *P* = 0.54), ASDAS (*r* = 0.02, *P* = 0.89), BASFI (*r* = −0.21, *P* = 0.23), ESR (*r* = 0.17, *P* = 0.33), or CRP (*r* = 0.15, *P* = 0.40). However, positive correlations of ESR and CRP with BASDAI, ASDAS, and BASFI were observed (Table 2). In addition, no significant correlation was found between age and serum sCD30 levels in healthy individuals (*r* = 0.09, *P* = 0.63; Figure 1(c)).

### 3.2. Discussion

AS is a chronic inflammatory disease that has a poor understood etiology and pathogenesis of disease. Due to the inconspicuous symptoms of AS, patients are often diagnosed many years after the original onset. The delay may be between 5 and 7 years, and as a result patients often experience irreversible damage of joints [19]. Therefore, there is an urgent need for early diagnosis and proper treatment of AS. However, the poor understanding of its cellular and molecular mechanisms strongly limit improvements in the clinical strategies for treating AS. Evidence to date suggests that imbalances of immune cells and the resulting aberrant cytokine profiles are involved in the pathogenesis of AS [1–3].

Several studies have demonstrated the potential value of measuring serum sCD30 levels in diagnosing and monitoring serious pathological conditions, including diseases due to malignancy, autoimmunity, and viruses [4, 9–14, 20]. However, only one clinical trial to date has assessed serum sCD30 levels in AS patients [21]. Based on the results from other institutions as well as our previous data in systemic lupus erythematosus (SLE) [4, 9–14, 19], a similar phenomenon was expected in AS patients. Our data showed a statistically significant increase in serum sCD30 levels in AS patients compared to age-matched healthy controls (Figure 1(a)). However, in a previous clinical study by Østensen et al., no significant difference in serum sCD30 levels was observed between AS patients and healthy controls (AS: *n* = 11; HC: *n* = 10) [21]. Compared to that study, the numbers of AS patients and HCs in our study were relatively higher, although only female participants were recruited in the previous study. In view of the fact that the number of males in the AS group of our study was notably higher than in the HCs group (Table 1) due to the male propensity of AS, we postulated whether this gave rise to the difference observed in the Østensen's study. Therefore, we compared serum sCD30 levels between healthy males (*n* = 12) and females (*n* = 20). We found no significant difference in serum sCD30 levels in the male and female HCs from our study (*P* > 0.05, Figure 1(b)), which was consistent with previous studies [22–24]. Furthermore, we found that serum sCD30 levels in healthy adults did not correlate with age (Figure 1(c)). Therefore, the evidence of a correlation between age and sCD30 levels remains controversial [23–26].

sCD30 was once identified as a marker of a T cell subtype that can produce Th2-type cytokines [23, 27, 28]. However, controversial results gradually emerged upon functional investigation of purified CD30+ T cells. For example, Pellegrini et al. reported that instead of a physiological marker of Th2 cells CD30 plays important roles in the regulation of the balance between Th1/Th2 cells by integrating Th1 and Th2 cell-specific cytokines and Bcl-2 expression. Inhibition of the CD30/CD30L interaction was proposed as a cause of equilibrium in the differentiation of Th0 to Th1 or Th2 cells. Moreover, studies have suggested that an abnormal increase of sCD30 levels can inhibit CD30 signals by blunting the CD30/CD30L interaction, which would subsequently lead to the Th1/Th2 imbalance [9, 23, 29–32]. Elevated sCD30 and soluble CD26 (sCD26) levels were previously shown by Mahmoudi et al. in patients with common variable immunodeficiency (CVID) and the authors proposed that the Th1/Th2 cell balance was impaired towards a Th1-like phenotype [32]. However, to the best of our knowledge, no study to date has investigated the Th1/Th2 cell balance induced by abnormal CD30 signaling in AS. Our present study did not investigate the numbers of CD30+ cells, other T cell subtypes, or their cytokines in both serum and involved tissues. Therefore, no conclusion can be drawn about whether sCD30 interferes with the Th1/Th2 cell balance and further studies are needed in this area.

Although significant correlations regarding serum sCD30 levels and disease severity-related indexes of various clinical disorders have been presented elsewhere [11, 12, 14], our results failed to find such a relationship in AS patients. Briefly, serum sCD30 levels did not correlate with BASDAI (*r* = −0.11, *P* = 0.54), ASDAS (*r* = 0.02, *P* = 0.89), BASFI (*r* = −0.21, *P* = 0.23), ESR (*r* = 0.17, *P* = 0.33), or CRP (*r* = 0.15, *P* = 0.40). However, all of the disease severity-related indexes showed remarkable positive correlations with ESR and CRP (Table 2), both of which are regarded as common markers of disease activity of AS [33, 34]. The most important limitation of the present study was the small sample size, which may have substantially attributed to the bias of the results. Moreover, the different disease stages of AS patients at the time of enrollment may have also led to unpredictable results. Nevertheless, a promising biomarker may yield important outcomes, even in a small cohort. Therefore, we propose that serum sCD30 is not a reliable biomarker in assessing disease activity of AS patients.

## 4. Conclusion

In summary, our results revealed an increase of sCD30 levels in AS patients, suggesting a potential role in the pathogenesis of AS. No correlation of sCD30 with BASDAI, ASDAS, or BASFI was observed in our study. Therefore, sCD30 cannot be used as a biomarker of disease severity and functional ability. A large prospective study is urgently needed to thoroughly investigate the precise roles of sCD30 in the pathogenesis of AS and the relationship among sCD30 and other indexes of disease severity.

## Figures and Tables

**Table 1:** Clinical and demographic information of AS patients and HCs.

	AS (*n* = 35)	HC (*n* = 32)	*P*
Age (mean ± SD year)	27.5 ± 8.7	27.9 ± 3.0	0.76
Male/female	33/2	12/20	0.007^*^
NSAIDs user (%)	0	—	
Glucocorticoid users (%)	2.9	—	
DMARDs user (%)	8.6		
BASDAI (mean ± SD cm)	4.2 ± 1.8	—	
ASDAS (mean ± SD cm)	3.3 ± 1.2	—	
BASFI (mean ± SD cm)	2.4 ± 2.1	—	
ESR (mm/h)	36.4 ± 33.7	—	
CRP (mg/L)	39.7 ± 40.7	—	

AS: ankylosing spondylitis; HCs: healthy controls; NSAIDs: nonsteroidal anti-inflammatory drugs; DMARDs: disease modifying antirheumatic drugs; BASDAI: bath AS disease activity index; ASDAS: ankylosing spondylitis disease activity score; BASFI: bath AS functional index; ESR: erythrocyte sedimentation rate; CRP: C-reactive protein.

^*^ *P* < 0.05.

**Table 2:** Correlations between clinical and laboratory values in AS patients.

	BASDAI (*r*)	ASDAS (*r*)	BASFI (*r*)	ESR (mm/h) (*r*)	CRP (mg/L) (*r*)
Serum sCD30 (ng/mL)	−0.11	0.02	−0.21	0.17	0.15
ESR (mm/h)	0.35^*^	0.68^**^	0.39^*^	—	0.79^**^
CRP (mg/L)	0.41^*^	0.81^**^	0.42^*^	0.79^**^	—

AS: ankylosing spondylitis; BASDAI: bath AS disease activity index; ASDAS: ankylosing spondylitis disease activity score; BASFI: bath AS functional index; ESR: erythrocyte sedimentation rate; CRP: C-reactive protein; *r:* correlation coefficient.

^*^ *P*< 0.05; ^**^ *P*< 0.01.
